# Symptoms and signs of colorectal cancer, with differences between proximal and distal colon cancer: a prospective cohort study of diagnostic accuracy in primary care

**DOI:** 10.1186/s12875-021-01452-6

**Published:** 2021-07-08

**Authors:** Knut Holtedahl, Lars Borgquist, Gé A. Donker, Frank Buntinx, David Weller, Christine Campbell, Jörgen Månsson, Victoria Hammersley, Tonje Braaten, Ranjan Parajuli

**Affiliations:** 1 Department of Community Medicine, UiT The Arctic University of Norway, 9037 Breivika, Tromsø, Norway; 2 Department of Health, Medicine and Caring Sciences, Linköping University, 58183 Linköping, Sweden; 3 Netherlands Institute of Health Services Research, Otterstraat 118, Utrecht, 3513 the Netherlands; 4 Department of General Practice, KU Leuven, Oude Markt 13, 3000 Leuven, Belgium; 5 Maastricht University, P.O. Box 616, Maastricht, 6200 The Netherlands; 6 Usher Institute of Population Health Sciences and Medical Informatics, University of Edinburgh, Edinburgh, EH8 9AG UK; 7 Department of Public Health and Community Medicine/Primary Health Care, University of Gothenburg, Box 100, 40530 Gothenburgh, Sweden; 8 Faculty of Nursing and Health Sciences, Nord University, P.O.Box 1490, 8049 Bodø, Norway

**Keywords:** Neoplasms, Colorectal cancer, Early diagnosis, Diagnostic accuracy, General practice, Family medicine, Primary Health Care

## Abstract

**Background:**

In an abdominal symptom study in primary care in six European countries, 511 cases of cancer were recorded prospectively among 61,802 patients 16 years and older in Norway, Denmark, Sweden, Netherlands, Belgium and Scotland. Colorectal cancer is one of the main types of cancer associated with abdominal symptoms; hence, an in-depth subgroup analysis of the 94 colorectal cancers was carried out in order to study variation in symptom presentation among cancers in different anatomical locations.

**Method:**

Initial data capture was by completion of standardised forms containing closed questions about symptoms recorded during the consultation. Follow-up data were provided by the GP after diagnosis, based on medical record data made after the consultation. GPs also provided free text comments about the diagnostic procedure for individual patients. Fisher’s exact test was used to analyse differences between groups.

**Results:**

Almost all symptoms recorded could indicate colorectal cancer. ‘Rectal bleeding’ had a specificity of 99.4% and a PPV of 4.0%. Faecal occult blood in stool (FOBT) or anaemia may indicate gastrointestinal bleeding: when these symptoms and signs were combined, sensitivity reached 57.5%, with 69.2% for cancer in the distal colon. For proximal colon cancers, none of 18 patients had ‘Rectal bleeding’ at the initial consultation, but three of the 18 did so at a later consultation. ‘Abdominal pain, lower part’, ‘Constipation’ and ‘Distended abdomen, bloating’ were less specific and also less sensitive than ‘Rectal bleeding’, and with PPV between 0.7% and 1.9%.

**Conclusions:**

Apart from rectal bleeding, single symptoms did not reach the PPV 3% NICE threshold. However, supplementary information such as a positive FOBT or persistent symptoms may revise the PPV upwards. If a colorectal cancer is suspected by the GP despite few symptoms, the total clinical picture may still reach the NICE PPV threshold of 3% and justify a specific referral.

**Supplementary Information:**

The online version contains supplementary material available at 10.1186/s12875-021-01452-6.

## Background

Globally, one in ten cases of new cancer is a colorectal cancer, and death from these cancers reaches almost the same proportion [1]. About half of colorectal cancer patients in the UK meet the UK National Institute for Health and Care Excellence (NICE) referral guidelines [2] and are diagnosed within three months after the first consultation in general practice [3]. However, the remainder often have atypical symptoms and longer diagnostic intervals [3]. Colorectal cancer is among the most frequently missed diagnoses in primary care [4]. This is a challenge for GPs in all countries.

We studied the relationship between abdominal symptoms and cancer in a cohort study with prospective registration of cancer. In two previous articles, we described the frequency of abdominal symptoms in general practice consultations, and what the general practitioner (GP) thought and did in relation to possible cancer [5]. The relationship between a symptom and a cancer diagnosis was also described [6]. This article focuses on symptoms and pathways to diagnosis in the subgroup of patients diagnosed with colorectal cancer.

## Methods

### Initial registrations

Between 25 February 2011 and 27 July 2011, GPs recruited through The Cancer and Primary Care Research International Network (Ca-PRI), registered 67,809 consecutive consultations with 61,802 patients 16 years and older in Norway, Denmark, Sweden, Netherlands, Belgium and Scotland. For initial registrations, participating GPs received a desktop workbook containing standardised daily registration sheets, one for each of ten working days (Additional file 1) with closed questions about symptoms recorded during the consultation [5]. Instructions were provided about how to record the different abdominal symptoms. For example, ‘Rectal bleeding’ should refer to bleeding “that can be observed or suspected macroscopically, either with red colour or melaena”. For patients with any specified abdominal symptom, non-specific symptoms and further diagnostic action were also recorded. Abdominal and non-specific symptoms listed were chosen from current medical literature related to cancer diagnosis in primary care.

### Follow-up

Eight months after each GP’s consultation period, GPs who had completed the initial registration sheets received forms for recording details of patients diagnosed with a new or recurring cancer after the consultation date (Additional file 2). The GPs were given their individual consultation dates and used their electronic records to identify these patients. The form was a simplified and revised version of a form used in two previous studies [7, 8]. All GPs were asked to supply anonymous information about the patients diagnosed with cancer during the follow-up period, whether or not they had presented symptoms during the initial survey. Free text comments accompanied multiple choice information about the diagnostic process, especially the role of clinical examination, laboratory tests ordered by the GP, and diagnostic procedures performed or ordered by the GP. Further symptoms, described in the medical record and originating between the consultation date and the date of diagnosis, were mainly reported in the GPs’ free text comments, especially answers to “Write in short form what primarily made you (or another physician) suspect cancer in this particular patient”. Most free text descriptions allowed transformation of “After consultation” symptoms into one of the pre-registered symptoms used in the initial registration forms. There were two special cases: “Abdominal pain” or “Acute abdomen” was registered as both ‘Abdominal pain, upper part’ and ‘Abdominal pain, lower part’, provided none of these symptoms had been recorded at consultation. Similarly, “Changes in bowel habit” was registered as both ‘Constipation’ and ‘Diarrhoea’. Two reminders were sent to GPs.

Sensitivity and specificity were calculated as the main measures of diagnostic accuracy, and positive predictive value suggested the probability of cancer. Only positive information from the questinnaires was used.

### Statistics

Statistical analyses were performed using SPSS, version 22. Fisher’s exact test was used to analyse differences between groups.

In addition to our own analyses, we added a pooled analysis of symptoms from our own material plus data from another primary care article [9] investigating symptom differences between proximal and distal colon cancer, and between colon and rectal cancer.

We used the STARD checklist when writing our report [10].

## Results

Four hundred ninety-three GPs completed the initial registration sheets. 315 (64%) GPs returned follow-up forms for one or more subsequent cancer patients. The last patient reported with cancer was diagnosed in April 2012. Among 511 patients with cancer, 65 patients had colon cancer and 29 had rectal cancer. Of these, 70 (14%), eight (12%) and six (21%) patients, respectively, had cancer recurrence.

### The most predominant abdominal symptoms recorded

Table 1 shows a great variety of pre-diagnostic symptoms recorded in the 511 cancer patients and in the subgroups of patients with subsequent colorectal cancer, new or recurrent, at the initial consultation or only at a later consultation. Among the 65 patients with colon cancer, 18 (28%) of the tumours were located in the right (proximal) colon, 26 (40%) in the left (distal) colon, while this was unclear for 21 (32%) patients. ‘Rectal bleeding’ and ‘Abdominal pain’, both upper and lower, were the most frequent symptoms: ‘Rectal bleeding’ was seen especially in the rectal cancer patients. ‘Constipation’, ‘Diarrhoea’ and the three non-specific symptoms were also frequently recorded. At least one symptom was recorded for 13 of the 18 patients with proximal colon cancer, and for 25 of 26 patients with distal colon cancer. Given any abdominal symptom recording, 19 of 55 (35%) colon cancer patients and 7 of 25 (28%) of rectal cancer patients also had non-specific symptoms.

**Table 1:** Number of patients with abdominal symptoms recorded during consultation, and between consultation and diagnosis of colon and rectal cancer

	**All cancer (*N *= 511)**	**Colorectal cancer (*N *= 94)**	**Colon (*N *= 65)**		**Proximal part of colon (*N *= 18)**		**Distal part of colon (*N *= 26)**		**Rectum (*N *= 29)**	
	**at consultation**	**at consultation**	**at consultation**	**after consultation**^a^	**at consultation**	**after consultation**^a^	**at consultation**	**after consultation**^a^	**at consultation**	**after consultation**^a^
Patient sex (Male / Female)	231/280	35/59	22/43		5/13		9/16		13/16	
Patient age (Median / Mean / Range)	71/69/28–96	75/71/39–92	73/71/39–92						75/70/49–86	
New < 180 days / New > 180 days / Recurrent	307/134/ 70	55/25/14	37/20/8						18/5/6	
**Abdominal symptoms**										
Abdominal pain, upper part	45	14	9	13	3	5	3	6	5	
Abdominal pain, lower part	37	14	9	15	3	5	3	8	5	
Constipation	22	13	7	6	2	1	2	2	6	2
Diarrhea	16	10	5	4			4	2	5	3
Distended abdomen, bloating	27	12	7				4		5	
Increased belching, flatulence	17	6	3		1		1		3	
Acid regurgitation	14	5	1				1		4	
Rectal bleeding	18	16	8	13		3	6	6	8	8
Unexpected genital bleeding	4									
Haematuria, macroscopic	7									
Increased urinary frequency	14	2	1				1		1	
Other abdominal problems	34	6	5	2	2		2		1	
Only one abdominal symptom	66	21	13	11	2	3	7	2	8	5
More than one abdominal symptom	63	23	13	17	3	5	7	8	10	2
Any abdominal symptom	129 (25%)	44 (47%)	26 (40%)	28	5	8	14	10	18 (62%)	7
No symptom recorded	382	50	39	11	13^b^	5	11^b^	1	11	4
**Non-specific symptoms (given at least one abdominal symptom)**										
Lack of appetite	26	10	7	3	1	1	3	1	3	
Unusual tiredness	25	9	6	3	1	1	2	1	3	1
Involuntary weight loss	18	6	3	7		3	3	2	3	3
Only one general symptom	24	5	4	6	1	3	1	1	1	2
More than one general symptom	19	9	6	3	1	1	4	1	3	1
Any general symptom	43 (33%)	14 (32%)	10 (38%)	9	2	4	5	2	4 (22%)	3

Patient sex and age, and time from consultation to diagnosis for new cases of cancer. Cohort study with 61,802 patients in primary care (2011–12). Distal colon: 25 sigmoideum + 1 descending. Proximal colon: 8 coecum + 1 appendix + 3 right flexure + 5 transversal + 1 proximal location not further specified. 21 cases of colon cancer did not have information about location. ^a^ After consultation, i.e. between consultation and diagnosis: Symptoms not already recorded at consultation for these patients. “Only one..”, “More than..” and “Any abdominal symptom” in this column count patients who had no symptom at consultation. ^b^ *P*-value distal/proximal part of colon = 0.07 (Fisher's exact test). “Abdominal pain” and “Acute abdomen” in free text have both been recorded as both upper and lower abdominal pain. “Changed bowel habit" recorded as both constipation and diarrhea

Bleeding from the intestines is shown in Table 2. Specificity for ‘Rectal bleeding’ reached 99.4%. PPV was 4.0% for colorectal cancer, but below 3% for the specific colorectal locations. However, the low number of patients in subgroups gave wide confidence intervals, and sex differences were not significant. For the subgroup of females 75 years of age or older, six patients had rectal bleeding and cancer at consultation, and PPV reached 11.1% for colorectal cancer. At consultation, none of the proximal colon cancer patients presented ‘Rectal bleeding’, while 27.6% of rectal cancer patients and 23.1% of distal colon cancer patients presented with this symptom. Sensitivity for colorectal cancer increased from 17.0% for ‘Rectal bleeding’ recordings at initial consultation, to 39.4% when symptoms recorded at subsequent consultations were taken into account. Adding information about positive test for occult blood in stool (FOBT) and/or the presence of anaemia, in patients without any recording of ‘Rectal bleeding’, sensitivity for one or more of these symptoms/signs increased to 57.5%; to 69.2% for cancer of the distal colon and to 38.9% for proximal cancer.

**Table 2:** ‘Rectal bleeding’ before diagnosis in colorectal cancer patients, with added sensitivity figures for patients with positive Faecal occult blood test (FOBT) and/or Anaemia. Cohort study with 61,802 patients in primary care (2011–12)

	N			Sensitivity (A)		PPV (A)				Sensitivity (B)		Sensitivity (C)		Sensitivity (D)	
	All	Males	Females	All	95% CI	All	95% CI	Males	Females	All	95% CI	All	95% CI	All	95% CI
Colorectal cancer	94	35	59	17.0%	10.8–25.9	4.0%	2.5–6.4	2.0%	5.1%	39.4%	30.1–49.5	47.9%	38.1–57.9	57.5%	47.4–67.0%
A	16	3	13												
B	37	13	24												
Colon cancer	65	22	43	12.3%	6.4–22.5	2.0%	1.0–4.0	0.7%	2.8%	32.3%	22.2–44.4	41.5%	30.4–53.7	55.4%	43.3–66.8
A	8	1	7												
B	21	6	15												
Colon, proximal cancer	18			-		-				16.7%	5.8–39.2	22.2%	9.0–45.2	38.9%	20.3–61.4
A	0														
B	3	1	2												
Colon, distal cancer	26			23.1%	11.0–42.1	1.5%	0.7–3.3	-	2.4%	46.2%	28.8–64.5	57.7%	39.0–74.5	69.2%	50.0–83.5
A	6	0	6												
B	12	1	11												
Rectal cancer	29	13	16	27.6%	14.7–25.7	2.0%	1.0–4.0	1.3%	2.4%	55.2%	37.6–71.6	62.1%	44.0–77.3	62.1%	44.0–77.3
A	8	2	6												
B	16	7	9												
Colorectal cancer (A), specificity:	Specificity 99.4% (95% CI 99.3–99.4), based on 385 patients with 'Rectal bleeding' without cancer, and 60,777 patients with no 'Rectal bleeding' and no cancer														

Faecal occult blood test (FOBT) positive: 6 patients with colon cancer and 2 patients with rectal cancer had positive FOBT, performed on clinical grounds but without any mention of rectal bleeding at or after consultation. Of the 6 colon cancer patients with FOBT + , 2 had cancer in the proximal colon, 3 in the distant colon, 1 unknown location. Anaemia': Recorded in 10 patients with colon cancer and 1 with rectal cancer when there was no recording of 'Rectal bleeding' at or after consultation. Of these, 1 patient with colon cancer and the patient with rectal cancer also had FOBT + . Of the 10 colon cancer patients with anaemia, 4 had cancer in the proximal colon, 3 in the distal colon, 3 unknown location. A. Number of patients with rectal bleeding and the specified form of cancer, and sensitivity, specificity and positive predictive value (PPV), based on consultation recordings for all patients. B. Number of patients with rectal bleeding and the specified form of cancer, and sensitivity, based on all recordings from consultation to diagnosis, in colorectal cancer patients. C. Sensitivity based on all recordings of 'Rectal bleeding' and/or FOBT +.  D. Sensitivity based on all recordings of 'Rectal bleeding' and/or FOBT + and/or Anaemia

Three of the other frequent symptoms have been analysed in Table 3. ‘Abdominal pain, lower part’, ‘Constipation’ and ‘Distended abdomen, bloating’ were less specific and also less sensitive than ‘Rectal bleeding’, and with a lower PPV. However, the symptoms were important for diagnosis in many patients, with little difference between proximal and distal colon cancer patients.

**Table 3:** Important symptoms, other than 'rectal bleeding', before diagnosis in colorectal cancer patients. Cohort study with 61,802 patients in primary care (2011–12)

Colorectal cancer				A						B	
	N			Sensitivity		Specificity		PPV		Sensitivity	
	All	Males	Females	All	95% CI		95% CI	All	95% CI	All	95% CI/Location
Abdominal pain, lower part'	94	35	59	14.9%	9.1–23.5	96.6%	96.4–96.7	0.7%	0.4–1.1	30.9%	22.4–40.8
'Abdominal pain, lower part' and cancer (A):	14	6	8								
'Abdominal pain, lower part' and cancer (B):	29	13	16								8 proximal, 11 distal, 5 unspecified, 5 rectal
'Abdominal pain, lower part', without cancer: 2084 patients											
No abdominal pain, lower part, no cancer: 59,078 patients											
'Constipation'				13.8%	8.3–22.2	98.9%	98.8–99.0	1.9%	1.1–3.2	22.3%	15.1–31.8
'Constipation' and cancer (A):	13	5	8								
'Constipation' and cancer (B):	21	7	14								3 proximal, 4 distal, 6 unspecified, 8 rectal
'Constipation', without cancer: 676 patients											
No constipation, no cancer: 60,486 patients											
'Distended abdomen, bloating'				12.8%	7.5–21.0	98.4%	98.3–98.5	1.2%	0.7–2.0	12.8%	7.5–21.0
'Distended abdomen, bloating', and cancer (A):	12	6	6								
'Distended abdomen, bloating', and cancer (B):	12	6	6								1 proximal, 4 distal, 2 unspecified, 5 rectal
'Distended abdomen, bloating', without cancer: 1011 patients											
No distended abdomen, bloating, no cancer: 60,151 patients											

A: Sensitivity, specificity and PPV based on consultation recordings for all patients. B: Sensitivity based on all recordings from consultation to diagnosis in colorectal cancer patients, and colorectal location

### The initiation of the diagnostic process

The majority of patients were symptomatic (Table 4). Fast track referral was used for 26% of patients with colon cancer and 38% of patients with rectal cancer. Urgent referral was used for 16% of colorectal cancer patients: for five patients with subsequent diagnosis of proximal colon cancer, two with distal colon cancer, five non-specified, and three patients with rectal cancer. A GP referred the patient in 51 cases (78%) of colon cancer and 26 cases (90%) of rectal cancer. The diagnostic process was initiated during the initial consultation in 3 of 18 cases (17%) with proximal location, and in 14 of 26 (54%) with distal location (*P *= 0,026); for rectal cancer in 12 of 29 (41%) patients. Hospital doctors initiated the search for cancer in 18% of colon cancer cases but in 28% with proximal location (not significant).

**Table 4:** Number of patients with symptomatic or asymptomatic initiation of the diagnostic process of colorectal cancer, in relation to location for colon cancer. Cohort study with 61,802 patients in primary care (2011–12)

Type of referral / Location of cancer	Colon Cancer			Rectal cancer
	Proximal (*n *= 18)	Distal (*n *= 26)	Unspecified (*n *= 21)	(*n *= 29)
A. Symptomatic patients (Colon 57, rectum 28)				
PC, ordinary referral (Colon 24, Rectum 13)	5	12	7	13
PC, fast track referral (Colon 17, Rectum 11)	4	7	6	11
PC, urgent referral (Colon 12, Rectum 3)	5	2	5	3
SC, no referral (Colon 4, Rectum 1))	2	2		1
B. Asymptomatic patients (Colon 8, Rectum 1)				
Screening (Colon 2, Rectum 1)		2		1
Incidentally (Colon 5)	2	1	2	
Do not know (Colon 1)			1	

*PC* Primary care, *SC* Specialist/hospital care

### The diagnostic role of the GP’s clinical examination, laboratory tests and diagnostic procedures, performed or ordered by the GP, and the seriousness of disease

Abdominal examination contributed to the diagnosis in about one third of the patients, for colon as well as rectal cancer (Table 5). Digital examination had a similar importance as abdominal examination in rectal cancer, and somewhat less for colon cancer patients. Proctoscopy/sigmoidoscopy contributed for 16% of patients, about equally for colon and rectal cancer. In 41% of patients, there was no diagnostic contribution from the clinical examination.

**Table 5:** The diagnostic role of clinical examination, laboratory tests and diagnostic procedures, performed or ordered by a general practitioner (GP), in colorectal cancer. Number of patients where these had diagnostic importance. Cohort study with 61,802 patients in primary care (2011–12)

	**Colon cancer (***n ***= 65)**	**Proximal location (***n ***= 18)**	**Distal location (***n ***= 26)**	**Unspecified (***n ***= 21)**	**Rectal cancer (***n ***= 29)**
**Clinical examination**					
Abdominal examination	19 (29.2%)	4 (22.2%)	7 (26.9%)	8 (38.1%)	10 (34.5%)
Digital rectal examination	13 (20.0%)	1 (5.6%)	7 (26.9%)	5 (23.8%)	11 (37.9%)
Gynecological examination	1			1	1
Proctoscopy/sigmoidoscopy	10 (15.4%)	1 (5.6%)	6 (23.1%)	3 (14.3%)	5 (17.2%)
Other examination	5		3	2	2
No diagnostic contribution from clinical examination	29 (44.6%)	11 (61.1%)	10 (38.5%)	8 (38.1%)	10 (34.5%)
Missing	4	2	1	1	1
**Laboratory tests**					
Haemoglobin concentration	17 (26.2%)	5 (27.8%)	5 (19.2%)	7 (33.3%)	2 (6.9%)
Erythrocyte Sedimentation rate	5	2		3	1
C-Reactive Protein	7	2	2	3	2
Test for occult blood in stool	17 (26.2%)	1 (5.6%)	8 (30.8%)	8 (38.1%)	4 (13.8%)
Cervical cytology	0				0
Prostate Specific Antigen	0				0
Urinary examination	0				0
Other	3			3	2
No diagnostic contribution from laboratory tests	29 (44.6%)	10 (55.6%)	13 (50.0%)	6 (28.6%)	18 (62.1%)
Missing	5	1	2	2	3
**Diagnostic procedures**					
X-ray	4	1	2	1	1
Ultrasound	2			2	1
Computer tomography	20 (30.8%)	7 (38.9%)	6 (23.1%)	7 (33.3%)	5 (17.2%)
Magnetic resonance	1	1			4
Upper GI Endoscopy	0				0
Coloscopy	47 (72.3%)	13 (72.2%)	19 (73.1%)	15 (71.4%)	20 (69.0%)
Cystoscopy	0				0
Other	2	1		1	3
None of the above procedures	6 (9.2%)	2 (11.1%)	3 (11.5%)	1 (4.8%)	1 (3.4%)
Missing	2		1	1	1

More than one examination/procedure could be recorded for one patient, where appropriate

The most important laboratory investigations were Haemoglobin (Hb) concentration and Faecal occult blood test (FOBT), mainly in colon cancer patients; one with proximal and eight with distal colon cancer. Laboratory tests did not contribute to diagnosis in 45% with colon cancer and in 62% with rectal cancer.

Coloscopy was diagnostically useful for about 70% of patients, for both colon and rectal cancers. Computer tomography (CT) examination contributed to the diagnosis in 31% of colon cancer patients and 17% of rectal cancers.

Colon cancer was localised in 45% of patients, and in 68% the treatment intention was curative. For rectal cancer, these figures were 34% and 76%, respectively. Table 6 shows that the number of symptoms had little relationship with how localised or spread the cancer was at the time of diagnosis. Among the 11 colon cancer patients with no symptoms, four had proximal, one distal and six unspecified cancer.

**Table 6:** Symptoms in relation to stage for colorectal cancer

Colon cancer (*n *= 65)		Stage			
At consultation	After consultation	Localised(*n *= 29)	Locally advanced(*n *= 18)	Distant metastases (*n *= 10)	Unknown or Missing(*n *= 8)
No symptoms	No symptoms	3	2	2	4
No symptoms	1–2 symptoms	13	9	2	3
1–2 symptoms	No symptoms	6	3	2	1
1–2 symptoms	1–2 symptoms	1		1	
3 + symptoms	No symptoms	4	4	3	
3 + symptoms	1–2 symptoms	2			
Rectal cancer (*n *= 29)		Stage			
At consultation	After consultaton	Localised(*n *= 10)	Locally advanced(*n *= 8)	Distant metastases (*n *= 9)	Unknown or Missing(*n *= 2)
No symptoms	No symptoms	1	1	2	1
No symptoms	1–2 symptoms	3	1	2	1
1–2 symptoms	No symptoms	2	3	2	
1–2 symptoms	1–2 symptoms	1	1		
3 + symptoms	No symptoms	2	2	2	
3 + symptoms	1–2 symptoms	1		1	

### Pooled analysis of symptoms

Differences between proximal and distal cancer, and between colon and rectal cancer, did not reach significance for our data. However, we found one previous general practice-based study of symptom differences between cancers in the proximal and the more distal parts of colon, reporting a scarcity of bleeding and change in bowel habits in proximal colon cancer (rectal bleeding: three cases of 22), compared with sigmoid (15 cases of 40) and rectal (40 cases of 57) cancers [9]. It was possible to pool data on rectal bleeding at referral from that study with our data on rectal bleeding at consultation. For pooled data, there were significant differences between proximal and distal colon cancer (*P *= 0.0038, two-tailed Fisher’s exact test), and between colon and rectal cancer (*P *< 0.0001). In both cases, there were more rectal bleeding in the more distal type of cancer.

## Discussion

### Main findings

Most abdominal symptoms investigated had a potential role in the detection of colorectal cancer. The high specificity of rectal bleeding for this form of cancer gives GPs a good reason for always seeking an explanation in patients with this symptom. A positive test for FOBT or iron-deficiency anaemia increases sensitivity for colorectal cancer. Maximising sensitivity is commonly considered as a wise diagnostic strategy as long as the lowering of specificity does not create an unacceptable false positive rate [11]. There is no clear distinction between definite alarm symptoms and lower risk symptoms, but abdominal pain and constipation stand out as among the more frequently presenting symptoms. Constipation combines high specificity with relatively high sensitivity and seems to be equally important for rectal cancer and colon cancer. Another important finding from the study is the relatively high specificity and sensitivity of ‘distended abdomen, bloating’, mostly recorded in rectal cancer and distal colon cancer.

Data about characteristics of symptoms stemming from the distal or proximal part of colon are based on relatively few cases, with wide confidence intervals for sensitivity and PPV. However, altogether they suggest that the GP should direct special attention towards the possibility of proximal colon cancer when symptoms are vague. The scarcity of rectal bleeding from cancers diagnosed in this location gives increased importance to the detection of possible bleeding through anaemia or FOBT. Unfortunately, FOBT has been shown to have slightly lower sensitivity for proximal than for distal colon cancer [12]. We did not ask whether guaiac-based tests or qualitative faecal immunochemical tests (FITs) were used. There is some evidence that the latter kind of test has higher accuracy than traditional tests, and combining FITs with the assessment of haemoglobin levels could improve sensitivity [13].

It should be noted that not all participating countries had established fast track pathways for colorectal cancer at the time of this study, Still, our study suggests that patients with proximal colon cancer seem to get less fast track referrals, have more urgent referrals, are perhaps more often discovered by hospital doctors and the diagnostic process is less frequently initiated by the GP at consultation. Scarcity or slow debut of symptoms, slow development of anaemia, fewer positive findings on clinical examination, insufficiency of sigmoidoscopy may contribute to this. Proximal colon cancer is less common than distal cancer, but the difference is perhaps smaller than many GPs think, 46 vs 54% in an Icelandic study [14]. Follow-up consultations in general practice may prove important as a start.

Clinical examination remains a mainstay of GP activity [15], and clinical findings as well as laboratory findings may each have had diagnostic importance for more than half of the colorectal patients. Diagnostic thinking mostly starts with symptoms which often give good diagnostic cues: however, a GP who routinely performs a simple but focused examination related to the medical history, may achieve new and sometimes unexpected insights. In one Danish study, abnormal laboratory values were associated with underlying cancer and could raise cancer suspicion [16]. Sensitivity of the total diagnostic picture may increase [8], i.e. a higher proportion of the GP’s patients presents with one or another type of ‘clue’ to diagnosis. Most GPs are aware that a negative digital rectal palpation has limited value in the detection of rectal tumours [17].

Colorectal cancer is among the cancers where early diagnosis has the highest impact on survival [18]. Patients in this study were most frequently diagnosed at a stage when there was still a hope of cure. The symptoms described often occur before the tumour has metastasised [19]. GPs thus have a crucial role in the colorectal cancer diagnostic pathways [20].

### Strength and limitations of the study

Despite the large cohort size, the number of colorectal patients was less than one hundred, and suggestive inferences rather than firm conclusions appear in this paper. However, some characteristics of colon cancer show consistent trends in our tables. The pooled analysis of symptom differences between different locations was not planned, and was undertaken only after we found the reference to the previous study. That analysis makes it more likely that the lack of significance for differences in our symptom data is due to the modest number of cases rather than differences not being real. It should be possible to outline diagnostic strategies based on the assumption that proximal colon cancer is indeed often rather symptom-poor.

The prospective nature of the follow-up implies that neither the patient nor the GP knew about the cancer diagnosis at the time of the initial symptom registration. In addition to the cross-sectional data recorded during the consultation, longitudinal data from medical records give a fuller picture of which symptoms the patient observed before diagnosis. Data from medical records are often incomplete, illustrated by the omission of a precise tumour location in many patients. However, this kind of symptom-based data probably has a high reliability [21], and a free text comment about what made the GP suspect cancer was missing for only one rectal cancer patient. For most patients in our study, it was possible to understand the approximate sequence of events leading up to the individual diagnosis.

Consecutive patients were registered sequentially and there was therefore no selection bias. The patient form was simple, with multiple choice answers and room for free text comments. For colorectal cancer, GPs seem to see most symptomatic patients before the diagnosis has been made. The proportion with new colorectal cancer (18.1%) was a little higher than for population based Norwegian figures (*P *= 0.016) [22]. There were more female patients diagnosed in our study population, relative to Norwegian incidence figures (*P *= 0.023 for colon cancer, *P *= 0.125 for rectal cancer). The number of patients is low, and we think the higher proportion of female patients may be coincidental.

With time, it becomes gradually less probable that there is a relationship between a symptom and subsequent cancer. In the second article from this study [6], we therefore limited analysis to patients with new cancer, diagnosed within six months after the consultation. This allowed for a more homogeneous group of patients. With fewer patients with a colorectal cancer diagnosis, we chose to include all patients in the study in the present article. Among patients with new cancer, 30% therefore were diagnosed more than six months after the consultation, as shown in Table 1.

### Strength and weaknesses in relation to other studies

For colorectal cancer, rectal bleeding, change in bowel habit and iron deficiency anaemia have been shown to have a PPV > 5% in higher age groups [23]. However, values this high for single symptoms are infrequent, and the UK NICE guidelines use a 3% risk threshold for recommending a suspected cancer pathway referral [24]. Based on Bayesian thinking, combinations of symptoms and signs may bring the cumulative PPV above 3% [25, 26]. Non-specific symptoms have low cancer relevance in themselves, but they gain in importance when associated with an alarm symptom [27].

Hamilton [28] suggested that referral decisions become more difficult for low-risk-but-not-no-risk symptoms. Our PPVs for abdominal pain, constipation, and distended abdomen, confirm this. However, in combination with other symptoms, PPV may rise. For single symptoms, a low PPV in younger age groups may increase with increasing age [29]. Previously published results from the current study population showed that GPs may have justifiable trust in their intuition-based cancer suspicion [5], recently confirmed in a meta-analysis [30]. Therefore, cancer pathway referral may be justified in many cases of patients presenting with low-risk symptoms. Our proportion of patients referred to fast-track diagnosis was slightly lower than that found for colorectal cancer patients in other studies [31, 32]. Diagnostic intervals decrease with dedicated cancer suspicion pathways [33, 34]. It is still uncertain whether different forms of fast track [35] lead to survival benefits in colorectal cancer [36, 37]. However, a Danish study of five common cancers including colorectal cancer, found that both the shortest and the longest diagnostic intervals were associated with higher 5-year mortality [38], the shortest due to already advanced disease at presentation. The authors concluded that this supports efforts to shorten the longest intervals through fast track pathways.

About 30% of cases of advanced colon neoplasia are not detected by sigmoidoscopy [39], while 36.5% of cases in a surgical study of malignant large bowel obstruction presentation had a right hemicolectomy [40]. Iron deficiency anaemia was the most common clue associated with missed opportunities for diagnosis of colorectal cancer [41]. A retrospective Icelandic study found that three of four patients with proximal colon cancer had anaemia, and they were more likely to be diagnosed incidentally than those with distal tumours [14]. Patients with non-specific but concerning symptoms were more likely than patients with alarm symptoms to be diagnosed at a later stage, and via an emergency presentation [42]. Rectal bleeding was less common in emergency presentations [43]. All these findings are in line with our findings of diagnostic clues to proximal colon cancer, i.e. less overt bleeding and more anaemia, and that diagnosis of proximal cancer is often more difficult.

### Implications for policy, practice and research

GPs should think about colorectal cancer as a group of diseases with three distinct locations. All locations have many similar presenting features, but location in the rectum, in the distal or in the proximal part of colon also have some distinct characteristics. Location in the proximal colon, i.e. caecum, appendix, ascending or transversal colon may have less typical symptoms but should raise awareness and be systematically assessed when symptoms are present but vague. The total clinical picture may then reach the NICE PPV threshold of 3% and justify a specific referral. To think of a proximal colon cancer when symptoms are limited is difficult, but referral may point to the possibility of colon cancer in general. Some of the GP’s cue may be to remember proximal cancer as a possibility when there is uneasiness and other explanations seem unsatisfactory. Such an approach could also contribute to earlier diagnosis for some more rare gastrointestinal tumours with unpredictable locations, like carcinoids or lymphomas.

Most abdominal symptoms merit the GP’s attention and efforts to collect more clinical and investigation evidence, in order to better assess the probability of a cancer diagnosis. However, an important proportion of cancer patients do not have the most common symptoms. Because the symptom presentation of colorectal cancer has such a wide spectrum, specific referral may be justified even when the clinical features are not among the common ones. Having an alarm symptom seems to give the highest probability of being referred to fast track routes, more so than the extent to which the GP considers the symptom to be serious [31]. Other cues, like positive clinical findings or test results, or ‘low-risk-but-not-no-risk’ symptoms in many cases should alert the GP’s suspicion of cancer and be the starting point of diagnostic work.

## Supplementary Information

**Additional file 1:** Initial registration form.

**Additional file 2:** Follow-up.pdf.

## Data Availability

The datasets used and/or analysed during the current study are available from the corresponding author on reasonable request.
