# Supplementary material for: Symptoms and signs of colorectal cancer, with differences between proximal and distal colon cancer: a prospective cohort study of diagnostic accuracy in primary care
Source: BMC Fam Pract. 2021 Jul 8;22:148. doi: 10.1186/s12875-021-01452-6 (PMC8268573; doi:10.1186/s12875-021-01452-6)
Supplement: Supplementary file 1 — Additional file 1: Initial registration form. [file 12875_2021_1452_MOESM1_ESM.pdf]

ABDOMINAL SYMPTOMS AND CANCER

GP number: 10001

Question 1-15: Duration in weeks
(State number of weeks 1-8, 9 if more than 8)

Question 32-34: One number (0-3) in each field

Please write: X or 7 Not: X or 7

Date: 23 08 10

Form number: 10

↦ To be completed only if abdominal symptom registered ↤

Abdominal symptoms (1–12): Duration in weeks, only one number 1-8, 9 if more than 8 weeks. General symptoms (13–16): Duration in weeks.

	Date of birth DD	MM	YY	Sex M	F	1 Abdominal pain, upper part	2 Abdominal pain, lower part	3 Constipation	4 Diarrhea	5 Distended abdomen, bloating	6 Increased belching, flatulence	7 Acid regurgitations	8 Rectal bleeding	9 Unexpected genital bleeding	10 Hematuria, macroscopic	11 Increased urinary frequency	12 Other abdominal problem	13 Lack of appetite	14 Unusual tiredness	15 Involuntary weight loss	16 None of these three (X)
1	11	04	42	X												3					X
2	20	06	57	X																	
3	29	01	45		X																
4	13	12	84		X																
5	03	10	66	X		4	4										9				X
6	23	10	79		X																
7	14	07	56		X																
8	09	04	61		X																
9	11	02	58		X																
10	17	05	89		X																
11	05	05	89		X																
12	27	12	49	X																	
13	16	04	66		X																
14	28	06	94		X																
15	30	10	66	X																	
16	17	04	73		X					5	5							2			
17	03	10	50	X																	
18	24	08	46		X																
19																					
20																					

First consultation? (17–18): One X. What organ system could be the site of somatic disease? (19–26): Minimum one X. Action decided during this consultation (27–31): Minimum one X. Cancer? (32–34): 0=not at all, 1=slightly, 2=medium, 3=strongly; One number in each. History (35–37): One X.

	17 First consultation for this episode	18 Not the first consultation	19 Stomach/duodenum	20 Large bowel	21 Rectum	22 Liver/gallbladder/pancreas/spleen	23 Urinary tract/male genitals	24 Uterus	25 Ovary	26 None of these seven	27 Lab tests, all kinds	28 X-ray/imaging	29 Referral/hospitalisation	30 Follow-up appointment with GP	31 None of these four	32 Symptoms suggest cancer…	33 Clinical findings suggest cancer…	34 My intuition suggests cancer…	35 Previous cancer? Yes	36 Previous cancer? No	37 Comments on the back
1		X					X				X			X		2	0	1	X		X
2																					
3																					
4																					
5	X			X		X	X				X			X		1	0	1		X	X
6																					
7																					
8																					
9																					
10																					
11																					
12																					
13																					
14																					
15																					
16	X		X	X					X		X	X		X		2	1	3		X	
17																					
18																					
19																					
20																					

”Abdominal symptoms and cancer”
Comments
Particularly for the questions 12, 27-29 and 35-36

Patient nr.	
1	35: Malignant melanoma, operated year 2000, considered cured
2	
3	
4	
5	12: Episodes of sudden, violent pain in urethra when urinating lasting 15-30 seconds, lithiasis?
6	
7	
8	
9	
10	
11	
12	
13	
14	
15	
16	
17	
18	
19	
20	
