# Supplementary material for: Symptoms and signs of colorectal cancer, with differences between proximal and distal colon cancer: a prospective cohort study of diagnostic accuracy in primary care
Source: BMC Fam Pract. 2021 Jul 8;22:148. doi: 10.1186/s12875-021-01452-6 (PMC8268573; doi:10.1186/s12875-021-01452-6)
Supplement: Supplementary file 2 — Additional file 2: Follow-up.pdf. [file 12875_2021_1452_MOESM2_ESM.pdf]

THE UNIVERSITY of EDINBURGH — AARHUS UNIVERSITY — KATHOLIEKE UNIVERSITEIT LEUVEN — nivel — Linköping University — UNIVERSITY OF TROMSØ

Follow-up form
Cancer patients

ABDOMINAL SYMPTOMS AND CANCER IN GENERAL PRACTICE

Dear GP-colleagues and participants!

We have now arrived at the follow-up of the prospective study “Abdominal symptoms and cancer in general practice”. Eight months ago you registered all your patient consultations during a 10-day period. We are very grateful for your participation. We are now at the stage of collecting follow up data on these consultations. Please complete and return this brief questionnaire. If the GP who collected the initial data is absent, we ask a colleague in the same practice to complete this form.

The dates you collected data on your patients are listed below. We ask you to check your electronic medical record for all of these patients. **Please look for any cancer diagnosis made after the consultation you had eight months ago, and report both new and recurrent forms of cancer.** For all patients we ask you to examine all available data to identify relevant patients. Please report all types of cancer.

If you DO NOT find any patients with a cancer diagnosis over the last eight months, we ask you to complete questions 1 - 11 in this questionnaire and return it in the pre-addressed envelope.

It’s likely you will find 1-2 new cases of cancer, new or recurrent. Exceptionally, 3-4 cases will be found. For the patients with cancer we ask you to complete one questionnaire for each patient with a cancer diagnosis.

We would now like you to kindly take the time to go through your patients at registration dates:

1. ____________	4. ____________	7. ____________	10. ____________
2. ____________	5. ____________	8. ____________	
3. ____________	6. ____________	9. ____________	

PS! Our budget allows us to pay €50 upon receipt of the completed questionnaire(s), and we ask you to return the enclosed bill with the questionnaire(s)

Kind regards,

Knut A. Holtedahl, M.D. Ph.D.
Professor, project leader
Phone: (+47) 77 64 48 87 / 997 49 370
E-mail: knut.holtedahl@uit.no

Børge Hart, M.D.
Ph.D. student, Project coordinator
Phone: (+47) 77 62 33 45 / 907 56 950
E-mail: borge.hart@uit.no

University of Tromsø, Dept. of Community Medicine, NO-9037 Tromsø, Norway
http://uit.no • Switchboard: (+47) 77 64 40 00 • Fax: (+47) 77 64 48 31
Project homepage http://www.uit.no/ism/absyca

Please turn

HOW MANY CANCER PATIENTS DID YOU FIND?

1. Date (*ddmmyy*) ☐☐☐☐☐☐
2. GP number ☐☐☐☐
3. I have reviewed all medical records for all patients registered during the initial ten day period: ☐ **YES** *Please mark with X*
4. Number of patients diagnosed with a new cancer between your initial registration period and now: ________ *(number)*
5. Number of patients diagnosed with recurrence of a previously treated cancer between your initial registration period and now: ________ *(number)*

If you found any cancers, new or recurrent, please fill in one patient form for each cancer patient.

Please include all types of cancer, not only abdominal cancers.
Exception: You do NOT need to report patients with Basal cell carcinoma of the skin (=rodent ulcer), or CIN (cervical intraepithelial neoplasia, where CIN3=cervical carcinoma in situ)

INFORMATION ABOUT THE GP

6. Sex of the GP. *M* ☐ *F* ☐
7. Age of the GP in years ☐☐
8. Who performed the initial data collection ('Time zero' data)?

   Only one x

   I did ☐

   Another GP in my practice did that ☐
9. I accept to be contacted by a researcher if additional information is required. ☐
10. I would like to have a scanned certificate of participation. ☐
11. I would like to have electronic copies of published articles. ☐

If yes to 9, 10 or 11, please write your mail address and telephone number here:

E-mail: ________________________________

Phone: ________________________________

THANK YOU VERY MUCH FOR YOUR COOPERATION

PATIENT FORM

1. Date of birth of the patient (*ddmmyy*): ☐☐☐☐☐☐

2. Sex of the patient: *M* ☐ *F* ☐

3. Year of first contact - for any reason - that you had with the patient (*yyyy*). ☐☐☐☐

4. Date, month and year for confirmed cancer diagnosis (*ddmmyy*). ☐☐☐☐☐☐
If you don't have exact date, write month & year like this: (*xxmmyy*). Histological date is best, imaging second best, clinical confirmation third.

A. WHAT KIND OF CANCER?

5. Current cancer type:

Only one x

New cancer: ☐
New recurrence of cancer: ☐

6. Current form of cancer; clinical diagnosis (including location if not evident):

____

____

7. Stage at diagnosis, from hospital report.

Only one x

Localised ☐
Locally advanced or regional spread ☐
Distant metastatic spread ☐
Do not know ☐

If there is a more precise staging, please write it here:

____

8. Histological diagnosis - please be as precise as possible.

____

____

9. Were there any metastases in the abdomen?

Only one x

Yes ☐
No ☐
Do not know ☐

10. If the hospital report states an ICD-diagnosis:

Please write the main ICD-code: **C** ☐☐☐

11. Diagnosis if any previously diagnosed cancer in this patient:

____

____

B. DIAGNOSTIC PROCESS

12. How was this cancer found?

Through symptomatic presentation: *Only one x*

-in primary care, with ordinary referral? ☐
-in primary care, with 'fast track' referral? ☐
-in primary care, with urgent referral? ☐
-in secondary care without referral from GP? ☐

Through non-symptomatic presentation:

-through screening? ☐
-incidentally through investigation for other disease? ☐

Do not know ☐

13. Was it you, who initiated cancer related diagnostics in this patient?

Only one x

Yes ☐
No, another GP initiated ☐
No, a hospital doctor initiated ☐
Do not know ☐

14. You (or a GP colleague) recorded a consultation with this patient approximately 8 months ago. Was this the time when the diagnostic process was initiated?

Only one x

Yes ☐
No ☐
Do not know ☐

15. Write in short form what primarily made you (or another physician) suspect cancer in this particular patient.

____

____

____

please turn

16. Concerning *clinical examinations* performed by you (or another GP) before diagnosis, please mark if one or more of the following contributed significantly toward diagnosis:

Mark with one or more x

- Abdominal examination ☐
- Digital rectal examination ☐
- Gynecological examination ☐
- Proctoscopy or sigmoideoscopy ☐
- Other examination ☐
- No diagnostic contribution from clinical examination ☐

What did you find, if any positive *clinical findings*?

____

17. Concerning *laboratory tests* ordered by you (or another GP) before diagnosis, please mark if one or more of the following contributed significantly toward diagnosis:

Mark with one or more x

- Haemoglobin concentration (Hgb) ☐
- Erythrocyte Sedimentation rate (ESR) ☐
- C-Reactive Protein (CRP) ☐
- Test for occult blood in stool (OBS) ☐
- Cervical cytology (Pap smear) ☐
- Prostate Specific Antigen (PSA) ☐
- Urinary examination ☐
- Other ☐
- No diagnostic contribution from laboratory tests ☐

What did you find, if any positive *test results*?

____

18. Did any of the following *diagnostic procedures* contribute to the diagnosis?

Mark with one or more x

- X-ray ☐
- Ultrasound ☐
- Computer tomography (CT) ☐
- Magnetic resonance (MRI) ☐
- Upper GI Endoscopy ☐
- Colonoscopy ☐
- Cystoscopy ☐
- Other ☐
- None of the above ☐

What was found, if any positive *findings* from any of the above?

____

C. OTHER IMPORTANT INFORMATION

19. At the time of the recorded consultation, did the patient have any symptomatic co-morbid conditions?

Only one x

- Yes ☐
- No ☐

If yes, can you please specify:

____

____

20. The intention of the cancer treatment just after the current diagnosis:

Only one x

- Curative ☐
- Palliative ☐
- Do not know ☐

21. Present state of the patient?

Only one x

- feeling well, no symptoms ☐
- stable disease ☐
- progressive disease ☐
- in the terminal phase ☐
- dead ☐

22. Your comments:

____

____

____

____

LUNDBLAD MEDIA AS – SVANEGODKJENT TRYKKSAK – 241 762
